# A nomogram for predicting the risk of male breast cancer for overall survival

**DOI:** 10.3389/fonc.2023.1068187

**Published:** 2023-08-04

**Authors:** Yahui Wen, Junjie Bai, Caihong Zheng, Jiameng Liu, Shunguo Lin, Hui Han, Chunsen Xu

**Affiliations:** ^1^ Fujian Medical University Union Hospital, Fuzhou, Fujian, China; ^2^ Department of Breast Surgery, Fujian Medical University Union Hospital, Fuzhou, Fujian, China; ^3^ Department of General Surgery, Fujian Medical University Union Hospital, Fuzhou, Fujian, China; ^4^ Department of Urology, Fujian Medical University Union Hospital, Fuzhou, Fujian, China; ^5^ Department of Breast Surgery, Women and Children’s Hospital, School of Medicine, Xiamen University, Xiamen, Fujian, China; ^6^ Breast Cancer Institute, Fujian Medical University, Fuzhou, Fujian, China

**Keywords:** male breast cancer, nomogram, predictive model, risk factors, SEER

## Abstract

**Background:**

Male breast cancer (MBC) is a rare disease, accounting for <1% of all male carcinomas. Lack of prospective data, the current therapy for MBC is based on retrospective analysis or information that is extrapolated from studies of female patients. We constructed a nomogram model for predicting the overall survival (OS) of MBC patients and verify its feasibility using data from China.

**Methods:**

Constructed a predictive model using 1224 MBC patients from the Surveillance, Epidemiology and End Results (SEER) registry between 2010 and 2015. The performance of the model was externally validated between 2002 to 2021 using 44 MBC patients from the Fujian Medical University Union Hospital. The independent prognostic factors were selected by univariate and multivariate Cox regression analyses. The nomogram was constructed to predict individual survival outcomes for MBC patients. The discriminative power, calibration, and clinical effectiveness of the nomogram were evaluated by the receiver operating characteristic (ROC) curve, and the decision curve analysis (DCA).

**Results:**

A total of 1224 male breast cancer patients were in the training cohort and 44 in the validation cohort. T status (*p*<0.001), age at diagnosis (*p*<0.001), histologic grade (*p*=0.008), M status (*p*<0.001), ER status (*p*=0.001), Her2 status (*p*=0.019), chemotherapy (*p*=0.015) were independently associated with OS. The diagnostic performance of this model was evaluated and validated using ROC curves on the training and validation datasets. In the training cohort, the nomogram-predicted AUC value was 0.786 for 3-year OS and 0.767 for 5-year OS. In the validation cohort, the nomogram-predicted AUC value was 0.893 for 3-year OS and 0.895 for 5-year OS. Decision curve analysis demonstrated that the nomogram was more benefit than the AJCC stage.

**Conclusions:**

We developed a nomogram that predicts 3-year and 5-year survival in MBC patients. Validation using bootstrap sampling revealed optimal discrimination and calibration, suggesting that the nomogram may have clinical utility. The results remain reproducible in the validation cohort which included Chinese data. The model was superior to the AJCC stage system as shown in the decision curve analysis (DCA).

## Introduction

Breast cancer is one of the most common malignancies worldwide for women. However, male breast cancer (MBC) is a rare disease, accounting for <1% of all male carcinomas (1). Due to the lack of data on risk factors, prognostic value, and treatment options related to MBC, the therapeutic patterns for male breast cancer that clinicians recommended are based on female breast cancer (2, 3).

However, whether the management of female breast cancer (FBC) can be used as a reference for MBC is still controversial. Some studies have concluded that MBC and FBC are two completely different types with different biological behaviors and should be treated differently (4, 5).

Therefore, a personalized prediction model is required for patients with male breast cancer. A nomogram is a simplified numerical model for statistical predictions that combines different independent factors (6–8). However, can the model built using the Surveillance, Epidemiology, and End Results (SEER) database be applicable to the Chinese? Few articles have been published on this subject.

In our study, a nomogram model was constructed by the SEER database for predicting the overall survival (OS) of MBC patients. Further, it was investigated whether the model was also applicable to the Chinese population.

## Materials and methods

### Patient selection and data collection

Data were acquired from the open-access, authoritative database of the SEER Program. Launched in 1973 by the United States Centers for Disease Control and Prevention and National Cancer Institute, the SEER database includes information on patients with endocrine, respiratory, digestive system, and other tumors, and covers approximately 34.6% of the population in the United States. The training cohort data used in this study came from a public, anonymous database and did not require ethics committee approval or informed consent. The validation cohort data were approved, and informed consent was obtained from the ethics committee of Fujian Medical University Union Hospital.

**Figure 1:**
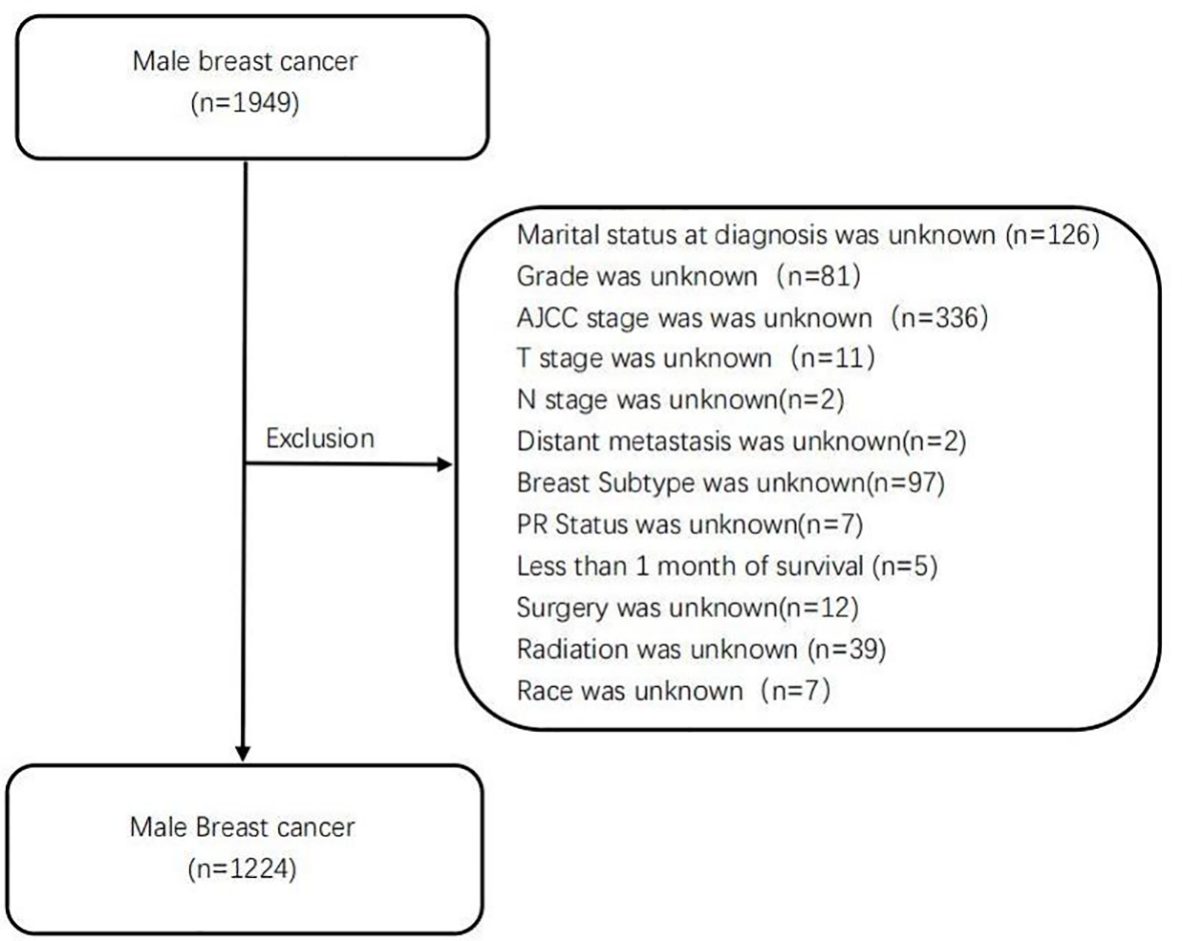
The flowchart of the selection for male breast patients in SEER database. SEER, Surveillance, Epidemiology, and End Results.

Training cohort data of MBC patients from 2010 to 2015 in the SEER database were extracted and screened by SEER Stat version 8.3.5 software. Validation cohort data from 2002 to 2021 in Fujian Medical University Union Hospital were extracted. Inclusion criteria were 1) pathologically diagnosed patients with breast cancer, based on the malignant behavior of International Classification of Diseases (ICD)-O-3 SEER site/histology validation code 8500/3, 2) male, and 3) complete clinicopathological and follow-up data. Exclusion criteria were 1) unknown important date, 2) with history of other types of cancer, 3) with less than 1 month of survival, and 4) diagnosis depends on biopsy/autopsy. According to the inclusion and exclusion criteria, cases meeting the criteria were gradually screened out, and 1,224 MBC patients were finally included in the training cohort. A total of 44 patients were included in the validation cohort. The study was not subject to review by the Institutional Review Board because we used unidentified, previously collected, and publicly available data. The flowchart of the male breast cancer selection is shown in **Figure 1**.

The clinicopathological information of patients in Fujian Medical University Union Hospital and the SEER database, including age, marital status, radiotherapy, chemotherapy, surgery, stage, grade, estrogen receptor (ER) status, progesterone receptor (PR) status, human epidermal growth factor receptor 2 (HER2) status, and subtype, was compared. Also, variables such as survival state and time were compared. Data from 1,224 patients extracted from the SEER database were used as the training cohort to analyze the independent influencing factors of MBC prognosis and establish a prediction model. The validation of the model was further demonstrated using the data of 44 patients from Fujian Medical University Union Hospital as the validation cohort.

### Statistical analysis

**Figure 2:**
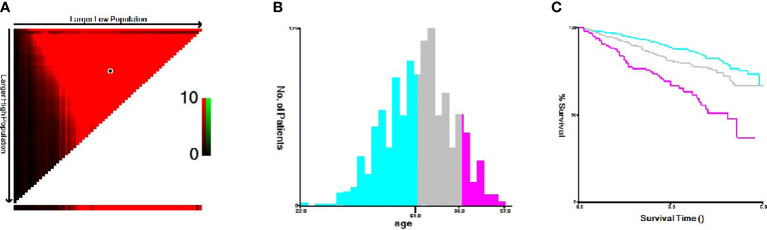
X-tile analysis of optimal cutoffs for age. **(A)** X-tile plot of the age training cohort. **(B)** Cutoffs are highlighted with histograms of the entire cohort. **(C)** Different prognoses determined by cutoffs are shown with Kaplan–Meier plots (age ≤ 64 years = blue, age 65–80 years = gray, and age >80 years = magenta).

Demographic and clinical characteristics were summarized using descriptive statistics. Categorical variables were reported as whole numbers and proportions, and continuous variables were reported as medians with standard deviation (SD). Pearson’s *χ* ^2^ test and Fisher’s exact test were used for categorical variables, and the Mann–Whitney U test was used for rank variables to compare the baseline characteristics of the training cohort and the validation cohort. The Kaplan–Meier method was used to describe the OS curve, and the log-rank test was used to evaluate the survival differences of distinct subgroups of each variable. The cutoff age for male breast cancer was determined by the X-tile procedure at 64 to 80 years (**Figure 2**). Patients were divided into three groups for further analysis (age ≤ 64, 65–80, and >80 years). Significant variables were screened by Cox univariate analysis, and variables with *p* < 0.1 in univariate analysis were included in the multivariate Cox proportional hazards model. The above statistical analyses were performed with IBM SPSS Statistics 26.

The prediction performance of the nomogram was internally verified by 1,000 resampling using the bootstrap method. The discrimination degree of the model was evaluated by the consistency index (concordance index (C-index)), receiver operating characteristic (ROC) curve, and area under the curve (AUC), and the model was detected by drawing the calibration curve. Degree of calibration was performed to ensure that the model is accurate and reliable. Decision curve analysis (DCA) was used to evaluate the overall survival of the nomogram compared with American Joint Committee on Cancer (AJCC) staging. Test level α = 0.05 (two-tailed). The above statistical analyses were performed with R 4.1.0 software.

## Results

### Patient characteristics

**Table 1** depicts the baseline characteristics of patients including training cohort (n = 1224) and validation cohort (n = 44). Pearson’s *χ* ^2^ test, Fisher’s exact test, and Mann–Whitney U test were used to compare the baseline characteristics of the training cohort and validation cohort. Marital status (*p* = 0.010), T status (*p* = 0.003), M status (*p* = 0.016), grade (*p* = 0.001), ER status (*p* < 0.001), subtype (*p* < 0.001), and radiotherapy (*p* < 0.001) were significantly different between the training and validation cohorts, which might be attributed to the difference of race. The average age of patients in the training cohort and validation cohort was 65.35 ± 12.24 and 58.57 ± 11.38 years, respectively, and the difference was statistically significant.

**Table 1:** Demographics and clinical characteristics of male breast cancer in training cohort and validation cohort.

Characteristic	Training Cohort, N (%)	Validation Cohort, N (%)	*X* ^2^	P-value
age	65.35±12.24	58.57±11.38		0.001
T status				0.003
T1	544 (44.4%)	30 (68.2%)		
T2	533 (43.5%)	11 (25.0%)		
T3	40 (3.3%)	2 (4.5%)		
T4	107 (8.7%)	1 (2.3%)		
N status				0.271
N0	667 (54.5%)	28 (63.6%)		
N1	384 (31.4%)	11 (25.0%)		
N2	109 (8.9%)	2 (4.5%)		
N3	64 (5.2%)	3 (6.8%)		
M status				0.016
M0	1144 (93.5%)	37 (84.1%)		
M1	80 (6.5%)	7 (15.9%)		
Stage				0.495
I	361 (29.5%)	19 (43.2%)		
II	578 (47.2%)	14 (31.8%)		
III	205 (16.7%)	1 (2.3%)		
IV	80 (6.5%)	10 (22.7%)		
Grade				0.001
Grade I、II	764 (62.4%)	33 (79.5%)		
Grade III	460 (37.6%)	9 (20.5%)		
ER status			24.252	<0.001
Positive	1191 (97.3%)	37 (84.1%)		
Negative	33 (2.7%)	7 (15.9%)		
PR status			3.386	0.066
Positive	1120 (91.5%)	35 (79.5%)		
Negative	104 (8.5%)	9 (20.5%)		
HER2 status			0.004	0.951
Positive	163 (13.3%)	6 (13.6%)		
Negative	1061 (86.7%)	38 (86.4%)		
Subtype			17.504	<0.001
HR+/HER2-	1040 (85.0%)	32 (72.7%)		
HR+/HER2+	154 (12.6%)	5 (11.4%)		
HR-/HER2+	9 (0.7%)	2 (4.5%)		
HR-/HER2-	21 (1.7%)	5 (11.4%)		
Surgery			2.143	0.143
Yes	1151 (94%)	39 (88.6%)		
No	73 (6.0%)	5 (11.4%)		
Chemotherapy			0.045	0.833
Yes	509 (41.6%)	19 (43.2%)		
No	715 (58.4%)	25 (56.8%)		
Radiotherapy			15.901	<0.001
Yes	368 (30.1%)	1 (2.3%)		
No	856 (69.9%)	43 (97.8%)		

ER, estrogen receptor; PR, progesterone receptor; HER2, human epidermal growth factor receptor 2; HR, hormone receptor.

### Univariable analysis and multivariable analysis

**Figure 3:**
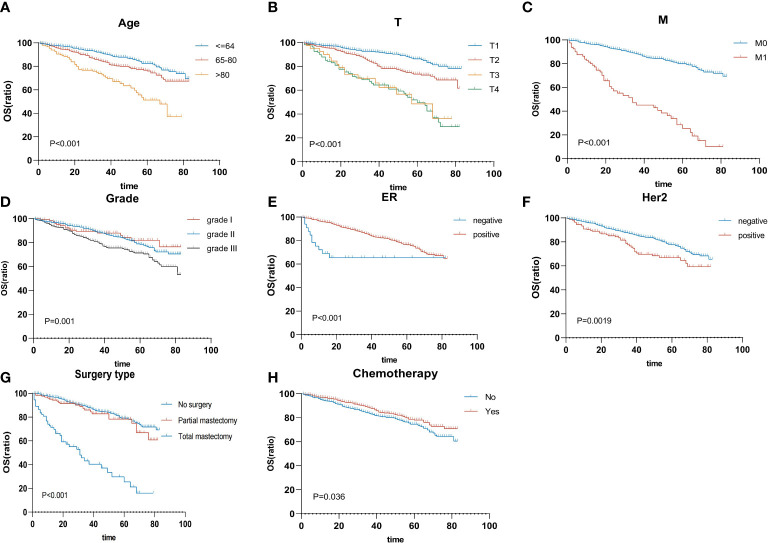
Overall survival rates are stratified by patient characteristics. Kaplan–Meier overall survival curves of the training cohort (*p* < 0.05) according to **(A)** age, **(B)** T status, **(C)** M status, **(D)** grade, **(E)** ER status, **(F)** HER2 status, **(G)** surgery type, and **(H)** chemotherapy. ER, estrogen receptor; HER2, human epidermal growth factor receptor 2.

On the univariable analysis (**Table 2**), age (*p* < 0.001), histologic grade (*p* < 0.001), T status (*p* < 0.001), N status (*p* < 0.001), M status (*p* < 0.001), AJCC staging (*p* < 0.001), ER status (*p* < 0.001), PR status (*p* = 0.019), HER2 status (*p* = 0.002), tumor subtype (*p* < 0.001), receipt of chemotherapy (*p* = 0.036), and surgery type (*p* < 0.001) were significantly associated with survival outcomes (all *p* < 0.05). On the multivariable analysis (**Table 2**) that included age (*p* < 0.001), histologic grade (*p* = 0.008), T status (*p* < 0.001), M status (*p* < 0.001), ER status (*p* = 0.001), HER2 status (*p* = 0.019), receipt of chemotherapy (*p* = 0.015), and surgery type (*p* = 0.001) were independently associated with survival outcomes (all *p* < 0.05). According to multivariable analysis, the Kaplan–Meier plot was used to show the differences in OS among these clinical benefits (**Figure 3**).

**Table 2:** Univariable and multivariable Cox regression analyses for OS in patients with MBC.

Variable	Univariable		Multivariable	
	HR (95%CI)	*p*-Value	HR (95%CI)	*p*-Value
Age		<0.001		<0.001
≤64 years	Ref		Ref	
65–80 years	1.53 (1.13–2.08)	0.006	1.93 (1.40–2.68)	<0.001
>80 years	3.32 (2.32–4.75)	<0.001	3.70 (2.45–5.60)	<0.001
Grade		0.001		0.008
I	Ref		Ref	
II	1.08 (0.64–1.81)	0.773	0.90 (0.53–1.52)	0.683
III	1.80 (1.08–3.00)	0.024	1.42 (0.84–2.40)	0.197
T status		<0.001		<0.001
T1	Ref		Ref	
T2	1.99 (1.44–2.76)	<0.001	1.70 (1.21–2.39)	0.002
T3	4.69 (2.69–8.17)	<0.001	3.03 (1.67–5.51)	<0.001
T4	5.02 (3.42–7.38)	<0.001	2.91 (1.87–4.55)	<0.001
N status		<0.001		0.107
N0	Ref			
N1	1.60 (1.18–2.16)	0.002		
N2	2.34 (1.57–3.51)	<0.001		
N3	1.99 (1.18–3.34)	0.009		
M status		<0.001		<0.001
M0	Ref		Ref	
M1	6.16 (4.47–8.47)		3.00 (1.86–4.84)	
Stage		<0.001		–
I	Ref		–	
II	1.47 (0.10–2.17)	0.052	–	–
III	2.71 (1.78–4.14)	<0.001	–	–
IV	9.42 (6.14–14.46)	<0.001	–	–
ER status		<0.001		0.001
Negative	Ref		Ref	
Positive	0.32 (0.17–0.59)		0.32 (0.17–0.62)	
PR status		0.019		0.556
Negative	Ref			
Positive	0.60 (0.39–0.92)			
HER2 status		0.002		0.019
Negative	Ref		Ref	
Positive	1.70 (1.22–2.36)		1.53 (1.07–219)	
Subtype		<0.001	–	–
HR+/HER2 −	Ref		–	–
HR+/HER2+	1.75 (1.25–2.46)	0.001	–	–
HR−/HER2+	2.31 (0.57–9.31)	0.24	–	–
HR−/HER2−	5.84 (2.97–11.47)	<0.001	–	–
Surgery type		<0.001		0.001
No surgery	Ref		Ref	
PM	0.19 (0.11–0.31)	<0.001	0.51 (0.28–0.93)	0.028
TM	0.14 (0.10–0.20)	<0.001	0.40 (0.25–0.63)	<0.001
Radiotherapy	0.90 (0.67–1.21)	0.476		
Chemotherapy	0.74 (0.56–0.98)	0.036	0.67(0.49–0.93)	0.015

ER, estrogen receptor; PR, progesterone receptor; HER2, human epidermal growth factor receptor 2; TM, total mastectomy; PM, partial mastectomy; OS, overall survival; MBC, male breast cancer.

### Nomogram construction and validation

**Figure 4:**
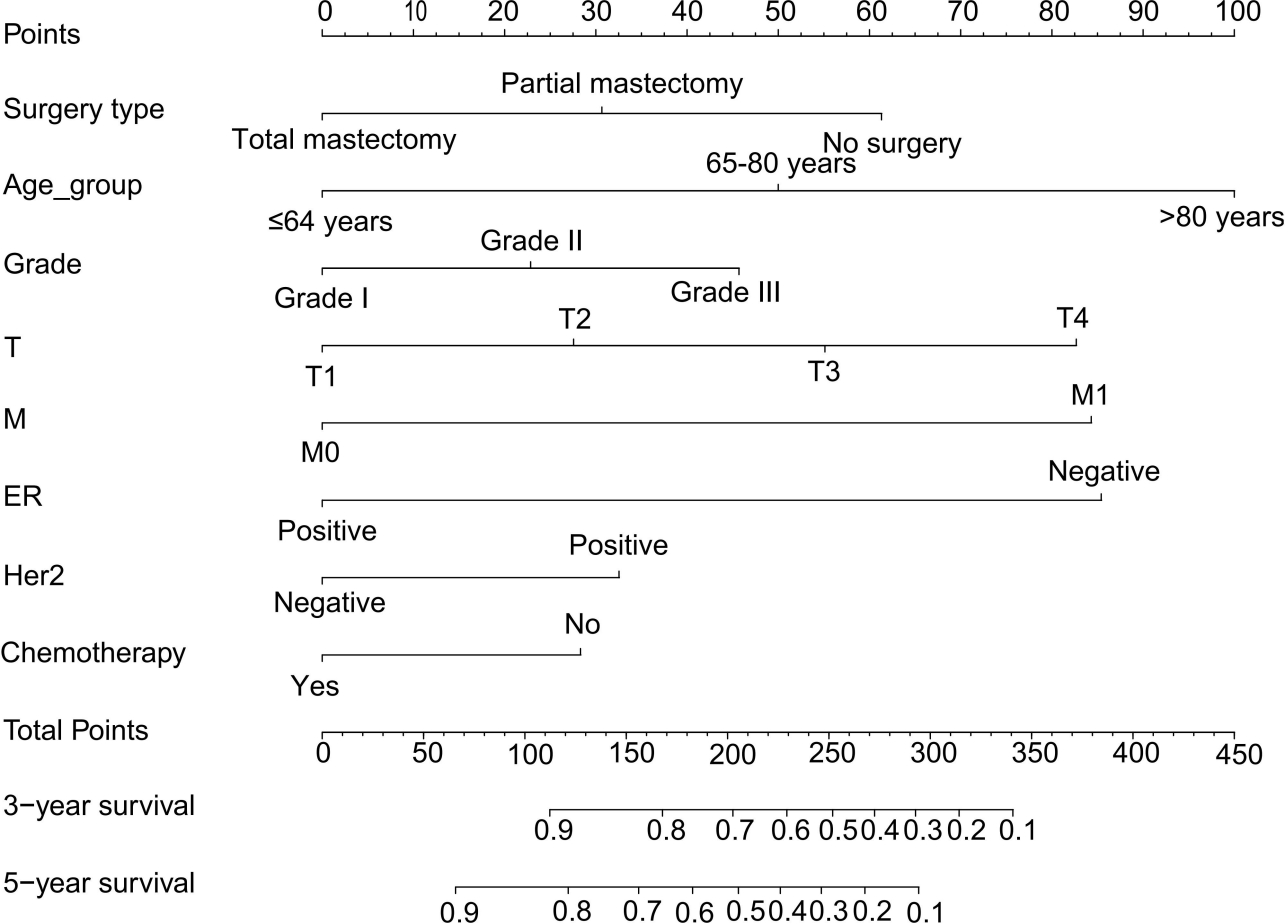
Nomogram predicting the 3-year and 5-year overall survival of MBC patients. Survival rates were determined by summing all scores and drawing a vertical line between the total score and the probability of survival scale. MBC, male breast cancer.

Multivariate-derived coefficients were used to develop a novel nomogram to predict male breast cancer 3-year overall survival and 5-year overall survival (**Figure 4**).

**Figure 5:**
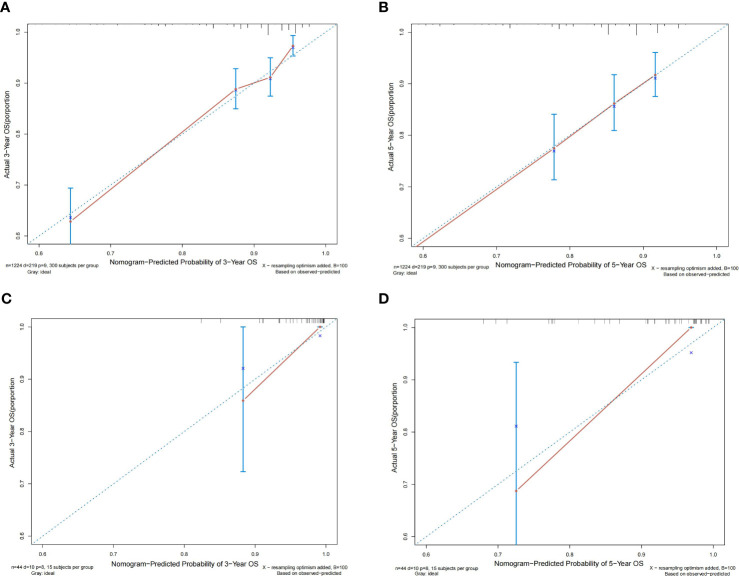
Calibration plots of the relationship between predicted probabilities and actual values based on nomograms. **(A, B)** Calibration curves for 3-year and 5-year overall survival in the training cohort. **(C, D)** Calibration curves for 3-year and 5-year overall survival in the validation cohort.

**Figure 6:**
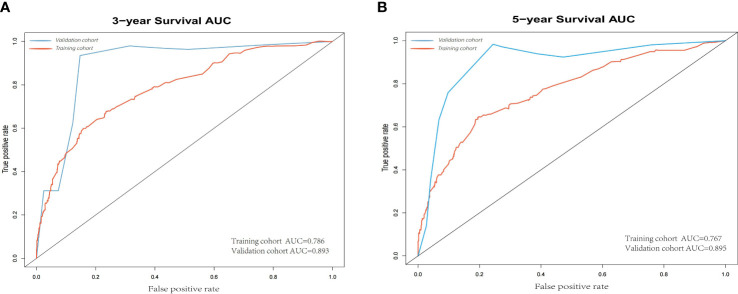
**(A)** 3-year ROC of OS nomogram using training and validation cohorts. **(B)** 5-year ROC of OS nomogram using training and validation cohorts. ROC, receiver operating characteristic; OS, overall survival.

According to the results, the nomogram contains age, histologic grade, T status, M status, ER status, HER2 status, receipt of chemotherapy, and surgery type. The nomogram illustrates that the ER status accounted for a vast majority of the proportion compared with other clinical features. The calibration curve of the nomogram showed high consistencies between the predicted and observed survival probability in both the training and validation cohorts (**Figure 5**). Perfectly calibrated models are indicated by dashed lines, and the results all show a good fit to the actual probabilities of the predicted probabilities. The ROC curves of the 3-year OS nomogram and 5-year OS nomogram for both the training and validation cohorts are shown in **Figure 6**. In **Figure 6A**, the 3-year OS AUC value was 0.786 in the training cohort and 0.893 in the validation cohort. In **Figure 6B**, the 5-year OS AUC value was 0.767 in the training cohort and 0.895 in the validation cohort.

**Figure 7:**
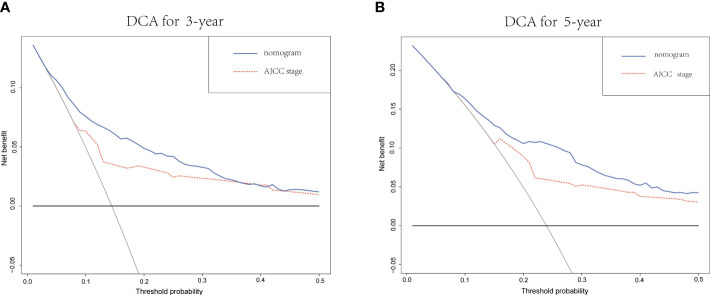
**(A)** The DCA of the nomogram and the AJCC stage system for 3-year OS in the training cohort. **(B)** The DCA of the nomogram and the AJCC TNM staging system for 5-year OS in the training cohort. DCA, decision curve analysis; AJCC, American Joint Committee on Cancer; OS, overall survival.

DCA curves showed that the nomogram could better predict the 3-and 5-year OS, as it added more clinical benefits compared with AJCC staging for all threshold probabilities in the training cohorts (**Figure 7**).

## Discussion

Breast cancer has become the most common malignancy in women worldwide, but breast cancer in men is still very rare. Due to its rarity, many clinical decisions have been informed and developed by the practice of female patients (9). However, MBC is considered to be a disease with distinct characteristics from FBC (5, 10). Meanwhile, an analysis from the National Cancer Database showed that overall survival rates for MBC remained lower than for FBC after adjusting for age, race, clinical, and treatment issues (11). Therefore, clinical characteristics and overall survival of MBC need to be further investigated.

From the baseline characteristics of MBC, the median age at the time of diagnosis of MBC is 65.35 ± 12.24 years, similar to a previous study (12). The majority of patients present with grade I or grade II disease (62.4%), ER-positive (97.3%), and less distant metastases (93.5%), compared with previous female studies (13, 14).

Traditionally, AJCC staging is the most general tool used to assess prognosis. It indicates the objective tumor load and metastasis status. However, the prognosis of tumors is composed of multiple biological and clinical factors. Current National Comprehensive Cancer Network (NCCN) and American Society of Clinical Oncology (ASCO) guidelines recommend the use of ER, PR, HER2, and Ki-67 status also as important prognostic factors in medical decision making. In addition to T, N, M, ER, PR, and HER2 status, in our Cox analysis, age, histologic grade, and whether or not to perform surgery and chemotherapy were also associated with OS. Therefore, it is necessary to establish a more comprehensive model to predict OS in MBC.

Previous research attempted to use predictive models for FBC on male breast cancer patients (15), but it was found that the predictive factors were not the same, possibly due to differences in the biological determinants of male and female breast cancer. Therefore, it is necessary for us to establish an independent predictive model based on data from male breast cancer.

In our study, in addition to surgery type, age, T status, M status, and histological grade, the expression status of ER and HER2, as well as the use of chemotherapy, also play important roles in the prognosis of MBC. It is noteworthy that N status was found to be significant in our univariate analysis but lost its significance in the multivariate analysis when considering multiple factors. This finding deviates from previous research results (16, 17). It is possible that the lack of significance of the N stage in the multivariate analysis could be due to a small sample size of male breast cancer cases included in our study.

It is worth noting that radiotherapy does not improve OS in MBC (*p* = 0.476). In previous studies of FBC, radiotherapy did improve local relapse in breast cancer patients, but whether radiotherapy improves OS remains controversial (18, 19). There are still few relevant studies of MBC. According to Kaplan–Meier survival analysis, our research findings indicate that there was no statistically significant difference in survival rates between male breast cancer patients who underwent total mastectomy and those who underwent partial mastectomy. This is consistent with previous research (20), suggesting that surgical procedures may not significantly impact survival outcomes in male breast cancer. However, adjuvant radiotherapy after partial breast resection may have mitigated potential survival differences. Further research with larger sample sizes and controlled confounding factors are needed for confirmation.

China has the highest number of breast cancer cases, accounting for approximately 18.4% of global breast cancer cases (21). In our study, the median age of diagnosis in China showed different patterns from the United States: the median age of diagnosis in China was almost 7 years earlier than that in the United States. This gap is smaller than in previous studies of FBC (22). Additionally, other different MBC features were demonstrated in our results, such as a higher proportion of T1 status patients, a higher proportion of grade I and II patients, a lower ER positive proportion, and a lower proportion of radiotherapy. There are differences in follow-up duration and basic patient characteristics between the training and validation cohorts. However, based on the ROC curves, it can be observed that the model achieved good validation performance across different baselines. Nevertheless, it cannot be denied that the bias in validation results may be influenced by different baselines. Therefore, further validation on multiple datasets is necessary.

In this model, the DCA curve indicates that this nomogram model has better predictions when compared to the AJCC staging. A higher C-index and a relatively high uniformity of the calibration plots were also shown in the model. In addition, we validated it with single-center data in China. Although there are more differences between the validation cohort and the training cohort, it also shows better validation results when external validation is performed. As far as we know, this is the first study to build and verify a nomogram in MBC with the SEER database and China single-center data.

Inevitably, our study has some limitations. First, this is a retrospective study, in which selection bias is inevitable. Second, some important confounding prognostic factors were not available in the SEER database, which include the Ki-67 index (23) and BRCA1- and BRCA2-related mutations (24, 25). Third, due to the data being derived from a single center, there is a need for additional validation using data from multiple centers to further assess the model's reliability and generalizability.

## Conclusion

Male breast cancer has been neglected due to its rarity, resulting in fewer studies related to treatment and prognosis. In this study, we developed a clinical prognostic model that combines the prognostic characteristics of male breast cancer and validated it with Chinese male breast cancer data. The results showed that the prediction model is applicable to different ethnic groups.

## Data availability statement

The raw data supporting the conclusions of this article will be made available by the authors, without undue reservation.

## Ethics statement

Written informed consent was obtained from the individual(s) for the publication of any potentially identifiable images or data included in this article.

## Author contributions

Conception and design: YW and CX. Development of methodology: YW, JL, CZ, SL, HH, JB, and CX. Acquisition of data, analysis, and interpretation of data (e.g., statistical analysis, biostatistics, and computational analysis): YW, JB, and CX. Writing, review, and/or revision of the manuscript: YW and CX. Study supervision: SL and HH. Manuscript revision: YW, JL, and CX. All of the authors reviewed and approved the final manuscript.
